# Resection of giant ethmoid osteoma with orbital and skull base extension followed by duraplasty

**DOI:** 10.1186/1477-7819-6-110

**Published:** 2008-10-14

**Authors:** Ioannis Yiotakis, Anna Eleftheriadou, Evagelos Giotakis, Leonidas Manolopoulos, Eliza Ferekidou, Dimitrios Kandiloros

**Affiliations:** 1 Department of Otolaryngology, University of Athens, "Hippokration" Hospital, Athens, Greece

## Abstract

**Background:**

Osteomas of ethmoid sinus are rare, especially when they involve anterior skull base and orbit, and lead to ophthalmologic and neurological symptoms.

**Case presentation:**

The present case describes a giant ethmoid osteoma. Patient symptoms and signs were exophthalmos and proptosis of the left eye, with progressive visual acuity impairment and visual fields defects. CT/MRI scanning demonstrated a huge osseous lesion of the left ethmoid sinus (6.5 cm × 5 cm × 2.2 cm), extending laterally in to the orbit and cranially up to the anterior skull base. Bilateral extensive polyposis was also found. Endoscopic and external techniques were combined to remove the lesion. Bilateral endoscopic polypectomy, anterior and posterior ethmoidectomy and middle meatus antrostomy were performed. Finally, the remaining part of the tumor was reached and dissected from the surrounding tissue via a minimally invasive Lynch incision around the left middle canthus. During surgery, CSF rhinorrhea was observed and leakage was grafted with fascia lata and coated with bio-glu. Postoperatively, symptoms disappeared. Eighteen months after surgery, the patient is still free of symptoms.

**Conclusion:**

Before management of ethmoid osteomas with intraorbital and skull base extension, a thorough neurological, ophthalmological and imaging evaluation is required, in order to define the bounders of the tumor, carefully survey the severity of symptoms and signs, and precisely plan the optimal treatment. The endoscopic procedure can constitute an important part of surgery undertaken for giant ethmoidal osteomas. In addition, surgeons always have to take into account a possible CSF leak and they have to be prepared to resolve it.

## Background

Osteomas are relatively rare, slow-growing, osteogenic tumors. They are the most frequent benign neoplasm of the paranasal sinuses, usually originating in the frontal sinus and much less in ethmoid, sphenoid and maxillary sinus. As osteomas are usually asymptomatic, they are very often incidental radiographic findings, most authors agree that small lesions do not need surgery suggesting periodic imaging in order to follow the growth and allow intervention before the development of complications [1]. Ethmoid osteomas appear early, as the limited anatomical space results to complaining by the patient. Extension to the orbit and/or skull base is unusual. When osteomas expand into the orbital vault, they displace the orbital contents and give rise to adequate symptoms, like headache, and ocular symptoms, such as diplopia, exophthalmos and proptosis.

Surgery is the treatment of choice for symptomatic ethmoid osteomas, however, the approach is under discussion and depends on the extension and the occurrence of complications [2]. Traditional surgical approaches to the involved sinuses are through external frontoethmoidectomy, lateral rhinotomy or osteoplastic flap technique [3]. Technological advantages in endoscopic instrumentation expanded the use of endoscopic surgery for the management of ethmoid osteomas. Endoscopic transnasal resection is ideal for tumors confined to the ethmoid and nasal cavity. The main advantages of the method are the minimal soft tissue dissection, the absence of facial bony disruption, and the avoidance of a facial incision. The magnification and the different angled view, which are possible with the use of endoscopes, may facilitate the removal of osteoma, with minimal morbidity [4]. However, when osteomas are large and expanded in to the orbit and anterior cranial base, a combination of external and endoscopic technique are required, due to the limited access and visibility of endoscopy.

We report a case of a bulky ethmoid sinus osteoma, with anterior skull base and intraorbital expand, treated with a combination of endoscopic and external approach.

We also report the management of SCF linkage presented in the same patient, performing duraplasty with fascia lata.

## Case presentation

**Figure 1:**
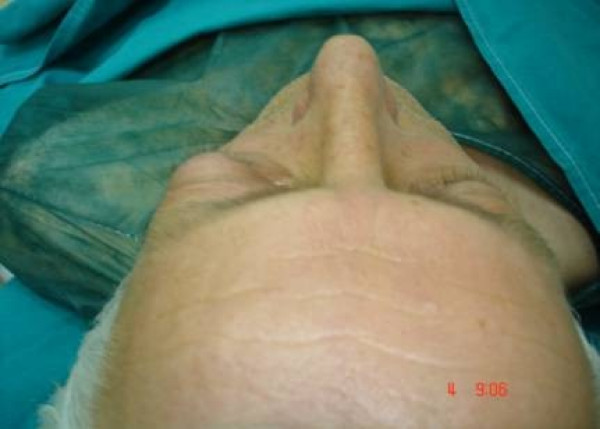
Preoperative photograph of the patient showing exophthalmos.

A 52-year-old man was referred to our department with a 3 year history of exophthalm, proptosis (Fig 1) and progressive visual impairment during the last 3 months.

**Figure 2:**
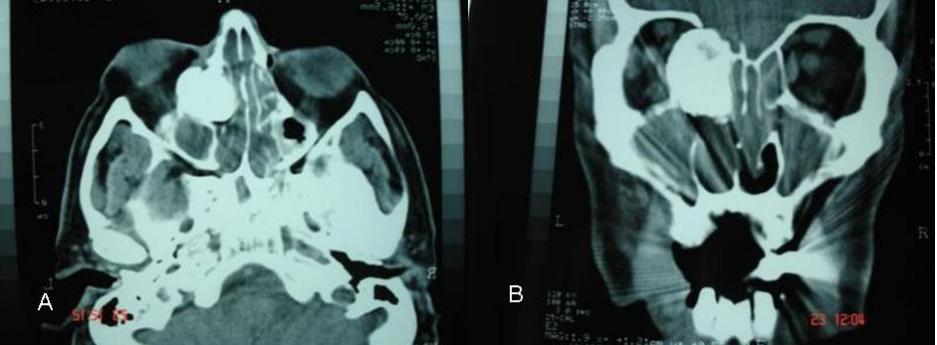
Preoperative computed tomography a) axial b) coronal.

**Figure 3:**
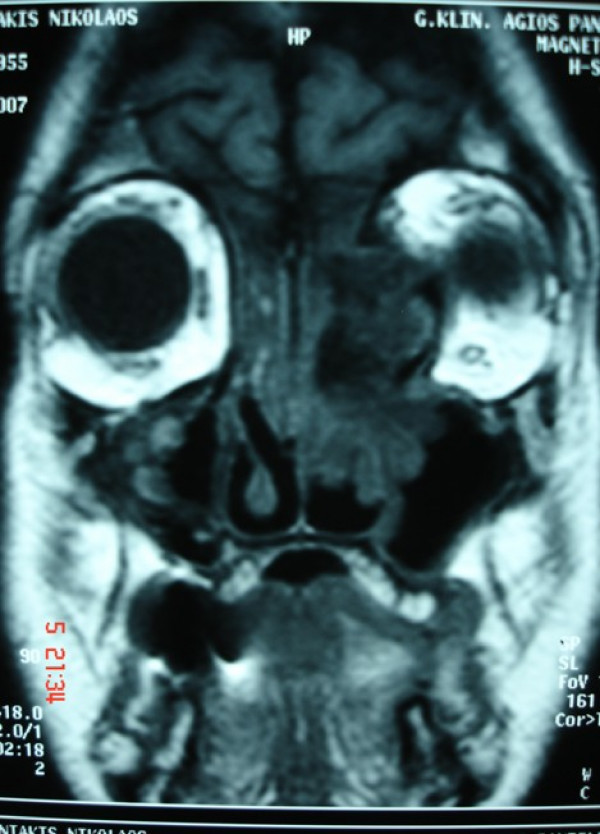
Preoperative coronal T1-weighted magnetic resonance image reveals a mass with lateral displacement of the left orbital contents and attachment of the tumor to the anterior skull base without intracranial involvement.

Assessment by means of coronal and axial computed tomography (CT) scan (Fig 2a, b) of the paranasal sinuses revealed a huge (6.5 cm × 5 cm × 2.2 cm) osteogenic lesion arising from the left ethmoidal labyrinth and expanded laterally into the orbit and cranially up to the anterior skull base. Left orbital contents were laterally displaced from the mass. Magnetic resonance imaging (MRI) depicted the compressed and diverted left optic nerve and showed that although osteoma was extremely close to the skull base and ethmoidal roof, there was not intracranial involvement (Fig 3). Nasal polyps were also found in both nasal cavities and both anterior and posterior ethmoid sinuses. Ophthalmologic exams showed proptosis of the left eye about 2.5 mm, diplopia on both gazes, motility limitation and exophtalmos. Visual field examination showed a small paracentral defect in the left eye. Visual acuity was 6/10 in the left side and 10/10 in the right side.

**Figure 4:**
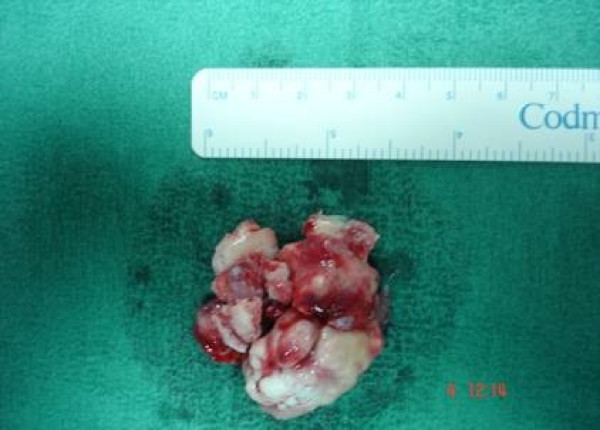
The residual specimen (after endoscopic endonasal drilling), removed via external incision.

Due to the size of the tumor, endoscopic removal was not feasible. Moreover, osteoma was broadly attached to the ethmoidal borders which did not allowed sufficient access to these borders using endoscopy. Hence, to create a better exposure, a combination of endoscopic endonasal technique with external approach carried out. The procedure was performed under general anesthesia; it began with a bilateral endoscopic polypectomy, followed by anterior and posterior ethmoidectomy and middle meatus antrostomy, using 0° and 30° endoscopes. Then, the size of the tumor was significantly reduced with the assistance of diamond drill. Afterwards, an external, non extensive "Lynch" frontoethmoidal incision was used around the left medial canthus in order to give access to the residual specimen. The mass was removed piecemeal. Lamina papyracea was in continuity with the osteoma. The orbit was gently shifted laterally, the osteoma was carefully detached from orbital periosteum and a piece of the osteoma was removed. Periosteum of the medial wall of the orbit was intact without any defect, so reconstruction was not necessary. Finally, the small remaining part of the osteoma was separated from the anterior skull base using a curved blunt elevator (Fig 4). After removal, a CSF leak was noticed and duraplasty was performed. The site of leakage was grafted with fascia lata and coated with bio-glu. After surgical intervention intra venous steroids were infused for about a week in order to diminish the periorbital ecchymoses and edema.

**Figure 5:**
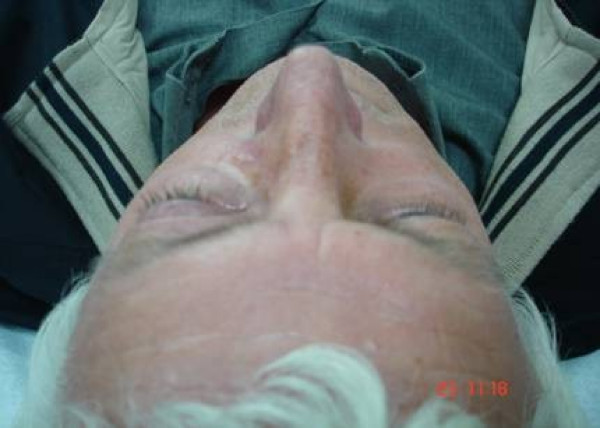
Postoperative photograph showing evident resolution of the exophthalmos.

**Figure 6:**
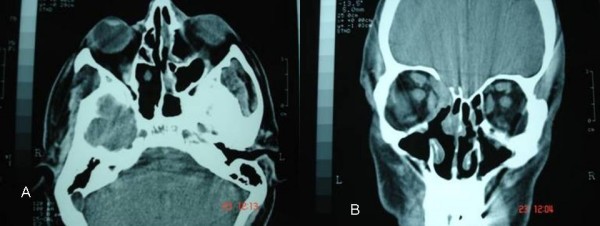
Postoperative computed tomographs a) axial and b) coronal.

Three months later, diplopia and proptosis had been resolved (Fig 5) and the patient recovered his visual acuity. Eighteen months after surgery, the patient remains without residue or recurrence (Fig 6a, b).

## Discussion

Although small frontoethmoidal osteomas are relatively frequent, giant osteomas are particularly rare findings in this region [5]. Lesions larger than 3 cm in diameter are considered giant tumors [6]. Due to the serious potential risks of surgery, osteomas of ethmoid sinus can be followed radiographically when they are asymptomatic. Surgery is performed only in the presence of symptoms and signs. Ethmoid osteomas expanded to the orbit and skull base are rare, and they are presenting with neurological and/or ophthalmologic complications like vision disorders, ptosis or headache. In the last cases excision becomes mandatory. Furthermore, surgery has been advocated for osteomas of the ethmoid sinus irrespectively of their size [7]. The surgical approach remains under discussion. Surgical techniques are adapted to different indications. For large ethmoid osteomas lateral rhinotomy, midfacial degloving, osteoplastic flap, external frontoethmoidectomy, and in selected cases, endoscopic excision, are discussed [8].

A detailed assessment of the margins of the tumor and definition of its relation with the surrounding structures is required in order to choose the most precise approach [9]. A CT scan is a fundamental tool that not only permits diagnosis but also allows the correct surgical approach to be planed. The three-dimensional CT scan is even described as a tool to define the extension of ethmoid osteomas [10]. In our case, careful analysis of CT scan in the axial and coronal view determined the size of the tumor and differentiated osteoma from soft tissue tumors or fibrous displasia. MRI imaging offered more exact evaluation of the margins of the lesion and finely revealed intraorbital extension but not intracranial invasion.

There are conflicting reports about the ability of an osteoma to recur after incomplete removal [11,12]. Nevertheless, we followed a surgical approach which led to complete removal of an osteoma. We realized that it was not possible to remove radically this huge tumor using endoscopic techniques because it was difficult to control all the tumor boundaries. However, endoscopic sinus surgery was of great help. Performing endoscopic polypectomy and middle meatus antrostomy, we gained visualization without bleeding and without any anatomical structure deformity. Then, via nasoendoscopic approach, the osteoma was drilled out in order to diminish the mass and profit better assess to the edges of the tumor. Although the mass was significantly reduced, detachment of the osteoma under the endoscopic route was not possible due to limited assess to the orbit and skull base. Thus, the remained part of the osteoma was dissected easily and safely with no extensive incision by an external Lynch approach around to medial canthus. Eroded dura was repaired with fascial graft.

There are several reports of successful removal of large ethmoid osteomas with intraorbital extension, treated endoscopically. Huang et al[13] have presented a case of ethoid osteoma extended in to the orbit, which was removed endoscopically after drilling and elevation. Naraghi et al[14] have described a case of large ethmoido-orbital osteoma dissected via endoscopic approach without drilling, with minimal complications. Apart from the much smaller size of the osteomas, in all these cases described above, serious visual or other complications were not quoted. In our patient, osteoma was giant (6.5 cm × 5 cm × 2.2 cm) and the presence of ophthalmologic complications demanded excision of the osteoma instantly.

It is worth mentioning, that this is not the first time that the coexistence of sinus osteoma with nasal polyps is reported. Since the etiology of the two entities is not fully investigated, it is possible that both of them are under the influence of similar etiological factors. In the past, post-traumatic and infectious causes have been discussed, and more recent studies advocate the role of developmental and genetic factors in the pathogenesis of both, nasal polyposis and sinus osteoma. [15].

## Conclusion

Endoscopic surgery meaningfully assists the removal of large osteomas of the ethmoids, minimizing soft tissue dissection and averting facial bony disruption. Surgeons may be faced during operative procedure with a CSF linkage. Therefore, they have to be prepared to repair it.

## Competing interests

The authors declare that they have no competing interests.

## Authors' contributions

IY, LM, and DK performed surgery, follow-up patient and helped in preparation of manuscript. AE prepared the draft of the manuscript. EG and EF helped to draft the manuscript. All authors read and approved the final manuscript.

## Consent

Written informed consent was taken from the patient for publication of this case report.
